# Synthesis and Properties of SPI/PLA–PCL Composite Microspheres

**DOI:** 10.3390/mi17050552

**Published:** 2026-04-29

**Authors:** Chunmei Wang, Qianshu Yu, Shuangsheng Zhang, Guoliang Zhang, Jiang Wu

**Affiliations:** School of Stomatology, Jiamusi University, Jiamusi 154000, China

**Keywords:** bone regeneration biomaterials, SPI/PLA–PCL composite microspheres, double emulsion–solvent evaporation method, osteogenic induction

## Abstract

According to the complementary advantages of the composites, the degradation rate, biological activity and physical and chemical properties of the composites were adjusted by using the hydrophilic and bioactive advantages of soy protein isolate (SPI) on the basis of toughening PLA by polycaprolactone (PCL). In this study, soy protein isolate/polylactic acid–polycaprolactone (SPI/PLA–PCL) composite microspheres were fabricated via double emulsion–solvent evaporation. SPI was introduced to regulate hydrophilicity, biodegradation, and bioactivity based on PCL–toughened PLA. The microspheres were characterized by SEM, EDS, FTIR, and XRD. Hydrophilicity, thermal stability, and degradation behavior were evaluated via water contact angle, TG/DTA, and in vitro degradation assays. Biocompatibility, hemocompatibility, and osteogenic activity were assessed through cell adhesion, hemolysis, CCK–8, ALP, alizarin red staining, and mineralization tests. Results confirmed the successful preparation of SPI/PLA–PCL microspheres. SPI incorporation enhanced hydrophilicity, degradation rate, and cell adhesion. The composite microspheres exhibited favorable thermal stability, hemocompatibility, biocompatibility, and osteogenic induction. The 50% SPI/PLA–PCL group performed optimally in cell proliferation, adhesion, ALP activity, and mineralization, demonstrating promising potential for bone tissue engineering applications.

## 1. Introduction

Maxillofacial bone defects caused by trauma, tumor resection, congenital deformities and periodontitis are still the main problems for clinicians. At present, the main materials used to repair bone defects are autogenous bone, allogeneic bone, metal materials, bioceramics, polymer materials and various composite materials [1]. Among them, the comprehensive properties of composites are better than those of single–component materials, which not only make up for their respective shortcomings, but also play a synergistic effect [2,3]. At the same time, the composite material has a wide range of sources, pollution–free and renewable, so it is a popular choice for the synthesis of bone tissue regeneration materials [4,5]. Therefore, it is an urgent problem for synthetic bone regeneration materials to choose two or more materials together to meet the performance of bone regeneration materials close to autogenous bone, and to overcome the shortcomings of autogenous bone [6,7].

Soy protein isolate (SPI) has the characteristics of rich source, renewable, biodegradable, biocompatible, hydrophilic and so on [8,9], and has been applied in food packaging, plastics, medical sutures, dressings, fibers, biofilms and so on [10,11]. However, soy protein isolate also has some shortcomings, such as poor water resistance, poor mechanical properties, etc. Therefore, solving the shortcomings of this material has become a hot topic in recent years [12,13].

Poly lactic acid (PLA) is a polyester of 2–hydroxypropionic acid, which can be degraded by enzymes and hydrolysis in vivo. This kind of material has good plasticity, degradability and biocompatibility. It is widely used in biomedical fields such as fracture internal fixation device, medical surgical suture and drug sustained release carriers. It is also one of the earliest bone tissue engineering materials [14,15]. However, there are still some shortcomings in its application as tissue engineering materials: ① Poor hydrophilicity. It is not conducive to cell adhesion [16,17]. ② Large brittleness. Polylactic acid (PLA) is a linear polymer with poor impact resistance [18,19]. ③ The degradation rate is slow, the degradation period is long, and it is not easy to control [20]. ④ It easily causes inflammation. Because the degradation products of PLA are acidic, it easily causes tissue cell inflammation [21,22]. In order to overcome the shortcomings of PLA as a tissue engineering material, and to prepare tissue engineering materials with high bioactivity, controllable degradation rate, excellent mechanical properties and good biocompatibility, it is often necessary to modify PLA raw materials [23,24].

Polycaprolactone (PCL) is a semi–crystalline linear aliphatic polyester, which is widely used as a highly hydrophobic and crystalline polymer. As a biomedical material, it has good biocompatibility, degradability, moderate mechanical strength [25], high flexibility, and its elongation at break is greater than 400% [26]. PCL and PLA are highly complementary in performance. Blending PCL with PLA to modify PLA can not only remedy the brittle nature of PLA, regulate its degradation kinetics, and alleviate local acidification during degradation, but also improve the processability of the composite, while preserving its biodegradability and enhancing its toughness and mechanical stability [27,28,29,30], laying a foundation for subsequent hydrophilic modification via SPI compounding.

In recent years, microsphere–based bone tissue engineering materials have attracted extensive attention owing to their unique advantages, including large specific surface area, favorable cell adhesion and nutrient exchange, minimally invasive injection into irregular bone defects, and precisely tunable structural and functional properties [31,32]. The double emulsion–solvent evaporation method is a mature and controllable microsphere fabrication technique, enabling uniform dispersion of multiple components within microspheres and endowing composites with stable physicochemical properties [33,34]. However, studies on PLA/PCL/SPI composite microspheres for bone regeneration are still insufficient, and systematic investigations into their structure–property relationships and biological activities remain lacking.

In this study, SPI/PLA–PCL composite microspheres were prepared by the multiple emulsion and solvent volatilization method. The possibility of using SPI/PLA–PCL composite microspheres as bone regeneration materials was evaluated through characterization analysis, physicochemical properties, biological properties (blood compatibility, biocompatibility, osteogenic inductivity) and other related tests.

## 2. Materials and Methods

### 2.1. Experimental Reagents and Instruments

#### 2.1.1. Experimental Reagents

The detailed information of experimental reagents is summarized in Table 1.

#### 2.1.2. Experimental Instruments

The detailed information of experimental instruments summarized in Table 2.

#### 2.1.3. Cells

Mouse embryonic preosteoblast (MC3T3–E1) were utilized as the cell model in the present study.

#### 2.1.4. Others

A wide range of experimental supplies and laboratory apparatus, such as well plates, tweezers, alcohol lamps and beakers, were additionally adopted in this work.

### 2.2. Preparation of Soybean Protein Isolate/Polylactic Acid–Polycaprolactone (SPI/PLA–PCL) Composite Microspheres

The microspheres were prepared by the multi–emulsion solvent volatilization method [35]:

(1) Oil phase: an electronic balance was used to weigh 2.0 g polylactic acid/polycaprolactone mixture (PLA: the PCL mass ratio was 85:15), and soy protein isolated with different mass fractions (SPI mass fraction 0%, 10%, 30%, 50%, 70%) was added with 80 mL dichloromethane, placed on a magnetic stirrer at 400 rpm, mixed until completely dissolved, and placed on a high–speed dispersion machine.
(2) Internal water phase: 1% (*w*/*v*%) NH_4_HCO_3_ aqueous solution. Weigh 0.25 g NH_4_HCO_3_ dissolved in 25 mL deionized water.
(3) Colostrum: Turn on the high–speed dispersing machine and stir at 3600 rpm. At the same time, add 1% (*w*/*v*%) NH_4_HCO_3_ aqueous solution to the oil phase at the rate of 2.5 mL/min, and continue to stir for 2 min after drip addition to form colostrum (W1/O).
(4) Double milk: the beaker containing 500 mL prepared 0.1% polyvinyl alcohol (PVA) aqueous solution is fixed on the paddle mixer, stirred at 400 rpm, and the colostrum is quickly poured into the PVA aqueous solution, and stirred for 10 h to form a double milk (W1/O/W2).
(5) Collection: Take off the beaker and stand for 10 min to precipitate the porous composite microspheres of soy protein isolate/polylactic acid/polycaprolactone, discard the supernatant, and transfer the porous composite microspheres into a 100 mL centrifuge tube.
(6) Water washing: The porous composite microspheres are cleaned with deionized water, and the centrifugal tube is turned upside down. After the porous microspheres are fully suspended, they are left for 10 min to precipitate the microspheres and discard the supernatant. This is repeated three times.
(7) Hydrolysis: Add the configured 0.1 MNaOH solution into the centrifugal tube equipped with microspheres, upside down, so that the porous microspheres are fully suspended; stand for 3 min, so that the microspheres are precipitated, and the supernatant is discarded. After three times cleaning with deionized water, the porous microspheres are obtained after standing.
(8) Sterilize the porous microspheres, freeze dryer dry, and wait for use. Divide the composite microspheres into control group, experimental group (4 groups), SPI content of 0% as the control group, SPI content of 10%, 30%, 50%, 70% as the experimental group.

### 2.3. Analysis and Characterization

#### 2.3.1. Field Emission Scanning Electron Microscope (FE–SEM)

FE–SEM is mainly used to observe the microstructure of samples. Because the sample is not conductive composite microspheres, we need to spray gold on the surface and then test it and perform energy spectrum (Energy Dispersive Spectrometer, EDS) analysis.

#### 2.3.2. X–Ray Diffraction (XRD)

The crystallinity of the sample was analyzed by XRD. The composite microspheres were put into the groove of the sample rack and the test conditions were: Cu–Kα radiation (voltage 36 KV, current 30 mA), in the 2θ zone 10°~60° range, maintain the scanning speed of 2°/min, step size of 0.02°.

#### 2.3.3. Fourier Transform Infrared (FT–IR)

Compound identification, molecular structure determination and functional group analysis of the sample can be performed by FT–IR to verify the chemical composition of the sample and to determine the substance. Potassium bromide tablet was used for sample preparation, and the scanning spectrum recording range was 4,000,400 cm^−1^. The prepared composite microsphere sample and dried potassium bromide were ground into powder with agate mortar, and the mixed powder was pressed into tablets and tested with FT–IR.

#### 2.3.4. Water Contact Angle Measurement

The freeze–dried composite microspheres were pressed by a tablet press and placed on a slide. The water contact angle of the samples was measured by a contact angle tester. Each group of samples was tested three times.

#### 2.3.5. Measurement of Porosity of Composite Materials

We used a MicroActiveAutoPoreV96002.03.00 mercury injection apparatus for porosity testing of porous composite microspheres. The freeze–dried porous composite microsphere with a certain mass (m_1_) was put into the dilatometer, and the mass (m_2_) was vacuum–sealed; the sample tube was put into the low–pressure chamber for low–pressure analysis. The analysis was completed when the pressure reached 0.2 MPa. The dilatometer was taken out of the low–pressure chamber and weighed again (m_3_). It was then loaded into a high–pressure station for high–pressure analysis, and automatically depressurized after reaching 200 MPa, which is a mercury removal process until the pressure returns to atmospheric pressure. The porosity of the porous composite microspheres was calculated by software based on the data of 3 weighings.

#### 2.3.6. Degradation Performance and pH Value Determination

The following is the recommended method according to ISO1378:2017 “Surgical Implants. Homopolymer copolymers and Blends on polylactic acid. In vitro degradation experiments” [36]: Take 50 mL plastic centrifuge tube, weigh 5 groups of composite microspheres (W0), and put in centrifuge tube, phosphate–buffer solution (PhosphateBufferedSaline, PBS) 30 mL, 37 °C choppy water bath, soaking period, record the pH of the solution with a pH meter. Samples were taken at a fixed time point on days 1, 3, 5, 7, 14, 21, and 28, cleaned with deionized water 3 times, freeze–dried, weighed (Wt) and recorded. The weight loss rate of microspheres before and after immersion is calculated as follows:

Weight loss rate of microspheres = (W0 − Wt)/W0 × 100%.

#### 2.3.7. In Vitro Mineralization

Scanning electron microscopy (SEM) was used to observe the mineralizing ability of the materials in vitro. All samples of the same quality were placed in simulated body fluid (SBF), which was changed every two days, and samples were taken out on the 7th and 14th day respectively, dried, and the mineralized morphology was observed by SEM.

#### 2.3.8. Differential Thermal Analysis

The thermal stability of composites can be evaluated by differential thermal analysis. The mass, temperature difference and heat change in the sample were recorded during the heating process. Using nitrogen atmosphere, the heating range is 30 °C~800 °C, and the heating rate is 10 °C/min.

### 2.4. Biocompatibility Test

#### 2.4.1. Hemolysis Test

Fresh blood was collected from volunteers using a vacuum collection vessel containing EDTA, and the blood was diluted with 0.9% NaCl solution (volume ratio of blood to 0.9% NaCl solution = 1:1.25). Five sets of nSPI/PLA–PCL (*n* = 0%, 10%, 30%, 50%, 70%) composite microspheres were placed in a 15 mL centrifugal tube, washed with steam and water three times, then washed three times with 0.9% NaCl solution—which was discarded for cleaning—and re–added with 5 mL 0.9% NaCl solution. After incubating at 37 °C for 10 min, 200 μL of blood diluent was added and incubated at 37 °C for 60 min. Then, 200 μL diluted blood was mixed with 5 mL triple steaming water as a positive control, and 200 μL diluted blood was mixed with 5 mL 0.9% NaCl solution as a negative control. After incubation, the centrifuge tube was removed and centrifuged at 1000 r/min for 10 min. The absorbance OD value of the supernatant at 545 nm was detected with an enzyme label. The calculation formula of hemolysis rate is as follows:

Hemolysis rate = (OD experimental group − OD negative control)/(OD positive control − OD negative control) × 100%

#### 2.4.2. Cell Experiments

##### Cell Culture, Passage, Cryopreservation and Resuscitation

When MC3T3 –E1 cells grow to 80–90% confluence at the bottom of the culture flask, subculture and cryopreservation are performed respectively. After trypsinization, the cells are inoculated into a new culture flask for continuous culture. After digestion, centrifugation and resuspension in freezing solution, the cells are cooled to −80 °C and stored. When recovering, the frozen cells at −80 °C are thawed in a water bath at 37 °C, centrifuged, resuspended, inoculated and cultured in an incubator. The culture medium is replaced the next day.

##### Liquid Preparation

Preparation of cell culture solution: DMEM medium 89 mL + fetal bovine serum (FBS) 10 mL + penicillin/streptin double antibody 1 mL; DMEM osteogenic induction medium was prepared: Dexamethasone 10 µg, vitamin C25 mg, β–sodium glycerophosphate 1.08 g, cell culture medium 500 mL; preparation of the extract: according to the recommended method of ISO10993–12:21 Biological Evaluation of Medical devices Part 12: Sample preparation and reference material standards [37], the extract was prepared with 0% SPI/PLA–PCL composite microspheres as an example. Cell culture medium was added in proportion (sample mass (g)/medium size (mL) = 0.2 g/mL). SPI/PLA–PCL composite microspheres were added into 1 mL cell culture medium, incubated at 37 °C, 5% CO_2_ and 95% humidity for 72 h, and centrifuged at 1000 rpm/min for 10 min. Remove the supernatant and filter it twice with a 0.22 μm filter membrane, label it and store it in a refrigerator at 4 °C, and use it within 7 days; control solution preparation: the cells cultured in cell culture medium were the negative control group. In addition, the culture hole with only culture medium but no cells was used as a blank control group.

##### Cell Proliferation Was Detected by CCK–8 Assay

Perform ultraclean table ultraviolet disinfection, remove the cells from the incubator, and observe the cell state under the microscope. The old medium was abandoned, cleaned twice with PBS, digested by pancreatic enzyme 90 s, and observed under the microscope; when the cells were round and did not float, the pancreatic enzyme digestive fluid was sucked out, 2 mL of medium was added to resuspend and mix the cells, and the cells were counted. MC3T3–E1 was inoculated into 96–well plates at 2.0 × 103/mL, 200 μL medium was added to each well, and each group of 5 compound wells, and the edges of the 96–well plates were filled with PBS, and cultured in a 37 °C, 5% CO_2_ constant temperature cell incubator for 24 h. After cell adhesion, the original media in blank control group, negative control group and experimental group were discarded, and 200 μL PBS was added to rinse 3 times. After the PBS was sucked out, 200 μL sample extract was added to each well of the experimental group. DMEM medium containing 10% FBS was added to the blank control group and the negative control group, and then the 96–well cell culture plates were placed in a constant temperature cell incubator at 37 °C and 5% CO_2_ for 1, 3 and 5 days; the solution was changed every 2 days, the old solution was discarded, the PBS was washed 3 times, and 100 μL medium containing 10 μLCCK–8 reagent was added to each well. Then, the 96–well cell culture plates were cultured in a constant temperature cell incubator at 37 °C and 5% CO_2_ for 1 h, and OD values of each well at 450 nm wavelength were detected by enzyme–labeling apparatus.

##### Cytotoxicity Was Detected by CCK–8 Assay

Perform ultraclean table ultraviolet disinfection, remove the cells from the incubator, and observe the cell state under the microscope. The old medium was abandoned, cleaned twice with PBS, digested by pancreatic enzyme for 90 s, and observed under the microscope; when the cells were round and did not float, the pancreatic enzyme digestive fluid was sucked out, 2 mL of medium was added to resuspend and mix the cells, and the cells were counted. MC3T3–E1 was inoculated on 96–well plates at 1.0 × 104/mL, 200 μL medium was added to each well, and each group of 5 compound wells, and the edges of 96–well plates were filled with PBS, and cultured in 37 °C, 5% CO_2_ constant temperature cell incubator for 24 h. After cell adhesion, the original media in blank control group, negative control group and experimental group were discarded, and 200 μL PBS was added to rinse for 3 times. After the PBS was sucked out, 200 μL sample extract was added to each well of the experimental group. DMEM medium containing 10% FBS was added to the blank control group and the negative control group, and the 96–well cell culture plates were cultured in a constant temperature cell incubator at 37 °C and 5% CO_2_ environment. Culture for 1, 3, and 5 days, replace the solution every 2 days, discard the old solution, rinse with PBS 3 times, add 100 μL medium containing 10 μLCCK–8 reagent to each well, and detect OD value of each well at 450 nm wavelength with enzyme–label analyzer. Cell survival rate (%) was calculated according to the absorbance values of each group, and the formula was as follows:

Cell survival rate (%) = (OD experimental group − OD blank control group)/(OD negative control group − OD blank control group) × 100%.

##### The Cell Adhesion and Growth Were Observed by Confocal Laser

MC3T3–E1 cells were cultured in 5 groups of materials/material extracts for 72 h, and stained with Rhodamine labeled Rhodamine phalloidin and DAPI reagent. The effects of composite microspheres on the adhesion and morphology of MC3T3–E1 cells were observed. Discard the old culture medium in the laser confocal dish, fix it with 4% paraformaldehyde fixing solution for 10 min, wash it with PBS 2–3 times, 3 min/time, add 0.5% TritonX–100 solution for 5 min, and wash it with PBS 2–3 times, 3 min/time. Add 100 nMRhodaminePhalloidin Rhodamine labeled phorus cyclic peptide solution to stain the cytoframework, incubate for 30 min in dark light, wash with sterile PBS 2–3 times, 3 min/time, add DAPI staining solution to stain the nucleus, incubate for 10 min in dark light, wash with PBS, and then observe under confocal laser microscope.

##### Determination of Alkaline Phosphatase (ALP) in Cells Cultured with Material Extract

MC3T3–E1 was inoculated into 6–well plates, and after cell adhesion for 24 h, the old medium was discarded and replaced with osteogenic induction solution of material sample extract of each group (2 mL per well), which was incubated in an incubator at 37 °C, 5% CO_2_ and 95% humidity. The solution was changed the next day, and ALP activity was detected at 7 and 14 days. Detection steps:

(1) MC3T3–E1 cells were inoculated into six–well plates and cultured in a constant temperature incubator at 37 °C and 5% CO_2_ for 24 h. The old medium was discarded and the extract containing osteogenic induction solution was added for further culture, and the solution was changed every other day.
(2) At the selected time points, 7 days and 14 days, the old medium was discarded, cleaned with PBS, digested by pancreatic enzyme for 23 min, observed under the microscope; when the cells were round and floating, the digestion was terminated by adding an equal amount of cell culture medium, and the cell suspension was transferred into the EP tube. Centrifuge at 1000 rmp/min for 5 min, discard the supernatant for precipitation, then add 1 mL PBS and gently blow, then centrifuge again at 1000 rmp/min for 5 min to discard the supernatant and leave the cell precipitation.
(3) Cell lysate 200 μL was added into the EP tube; lysate on ice for 30 min, centrifuge at 4 °C and 12,000 r/min for 10 min, and leave the supernatant for use.
(4) Add 0.5 mM of standard application liquid 4 μL, 8 μL, 16 μL, 24 μL, 32 μL and 40 μL to the standard holes of the 96–well plate respectively, and then add buffer liquid to each hole to make the standard hole liquid amount reach 100 μL, add 50 μL of the sample to be measured to the test hole drop, and add 50 μL buffer solution to the blank hole drop. Add 50 μL of prepared color developing substrate to the measuring hole and the blank hole. Mix well and incubate at 37 °C without light for 30 min. OD values of absorbance of each hole were determined by enzyme–labeled instrument (wavelength at 405 nm), and there were multiple holes in each group. The protein concentration of each group was measured by BCA kit, and the absorbance OD value of each hole was measured by enzyme–labeling instrument (wavelength at 520 nm).

##### Matrix Mineralization Staining and Semi–Quantitative Detection

MC3T3–E1 cells were co–cultured with nSPI/PLA–PCL composite microspheres (*n* = 0%, 10%, 30%, 50%, 70%) in mineralization induction medium for 7 and 14 days, the old medium was discarded, the PBS was washed three times, and the composite microspheres were transferred to the new pore plate, and an appropriate amount of 4% paraformaldehyde was fixed for 10 min. Wash with PBS 3 times, incubate with 0.2% alizarin red dyeing solution at 37 °C for 30 min, wash with PBS 3 times, and take pictures of microspheres under a type microscope. Then 10% cetylpyridine chloride was dissolved for 2 h, and the absorbance of the resulting supernatant was measured at 562 nm.

### 2.5. Statistical Analysis

The measurement data were expressed as Mean ± SD. SPSS22.0 software was used, *t*-test was used for data comparison between two groups, and one–way ANOVA test was used for data comparison between multiple groups. *p* < 0.05 was considered statistically significant.

## 3. Results and Discussion

### 3.1. Characterization of Soybean Protein Isolate, Polylactic Acid/Polycaprolactone (nSPI/PLA–PCL) Composite Microspheres

#### 3.1.1. SEM of nSPI/PLA–PCL Composite Microspheres

In this work, nSPI/PLA–PCL composite porous microspheres were fabricated via the double emulsion–solvent evaporation method [38]. Dichloromethane, which is immiscible with water and exhibits a low boiling point and high vapor pressure, was employed as the oil phase, in which PLA, PCL, and SPI were uniformly dissolved. A 1% NH_4_HCO_3_ aqueous solution acted as both the internal aqueous phase and porogen. Under high–speed homogenization, the internal aqueous phase was dropwise added into the oil phase to form a stable W/O primary emulsion [39]. Subsequently, the primary emulsion was introduced into the external aqueous phase containing PVA to construct a W/O/W double emulsion system [40]. With the gradual volatilization of the organic solvent, the polymer concentration in the oil phase continuously increased, triggering liquid–liquid phase separation and finally leading to the solidification and precipitation of nSPI/PLA–PCL composite microspheres [41]. Meanwhile, NH_4_HCO_3_ in the internal aqueous phase thermally decomposed to generate gas bubbles, which served as a key driving force for the formation of abundant internal pores.

**Figure 1:**
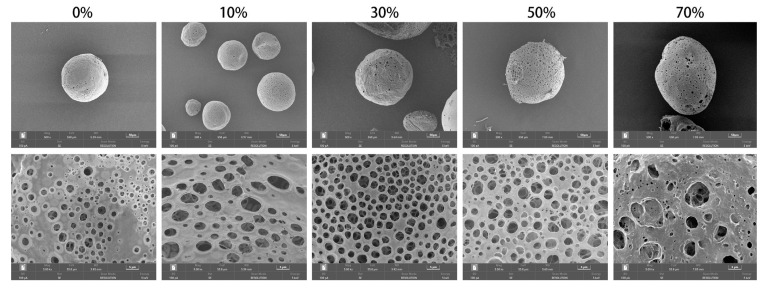
Scanning electron microscope image of nSPI/PLA–PCL composite microspheres.

As can be seen in Figure 1, the nSPI/PLA–PCL microspheres exhibited regular spherical or subspherical morphologies with relatively smooth surfaces. Abundant porous structures were observed both on the surface and inside the microspheres, which were favorable for cell adhesion, nutrient exchange, and tissue ingrowth in bone regeneration applications. With increasing SPI content, the particle size of the composite microspheres slightly increased and the surface became rougher [42]. This can be explained by the strong hydrophilicity of SPI, which increased the viscosity of the oil phase, slowed down solvent diffusion and emulsion homogenization efficiency, and disturbed the interfacial stability of the emulsion system, resulting in larger particle size and rougher surface morphology [43,44].

#### 3.1.2. EDS of nSPI/PLA–PCL Composite Microspheres

**Figure 2:**
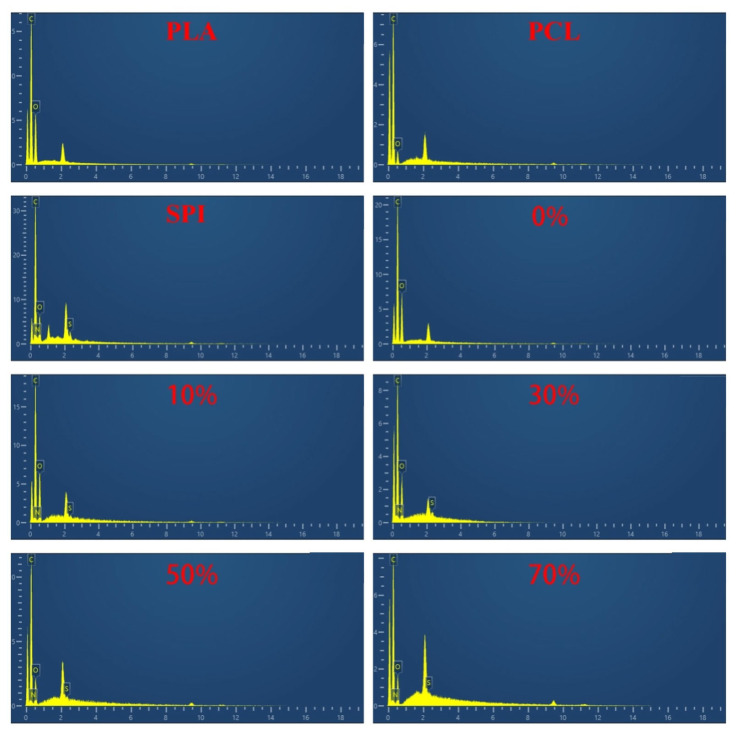
EDS spectra of pure PLA, pure PCL, pure SPI, and nSPI/PLA–PCL composite microspheres with different SPI mass fractions (0%, 10%, 30%, 50%, 70%).

To clarify the phase composition of nSPI/PLA–PCL composite microspheres, EDS characterization was synchronously performed at the identical observation field of view corresponding to SEM morphological characterization. Meanwhile, EDS spectra of pure PLA, PCL and SPI raw materials were supplemented for component comparison. As presented in Figure 2, pure PLA and PCL matrices are mainly composed of C and O elements, while SPI displays characteristic C, O, N and S elements. All SPI–loaded composite microspheres exhibit coexistent C, O, N and S elemental signals, verifying the successful immobilization of SPI inside composite microspheres.

#### 3.1.3. FTIR of nSPI/PLA–PCL Composite Microspheres

**Figure 3:**
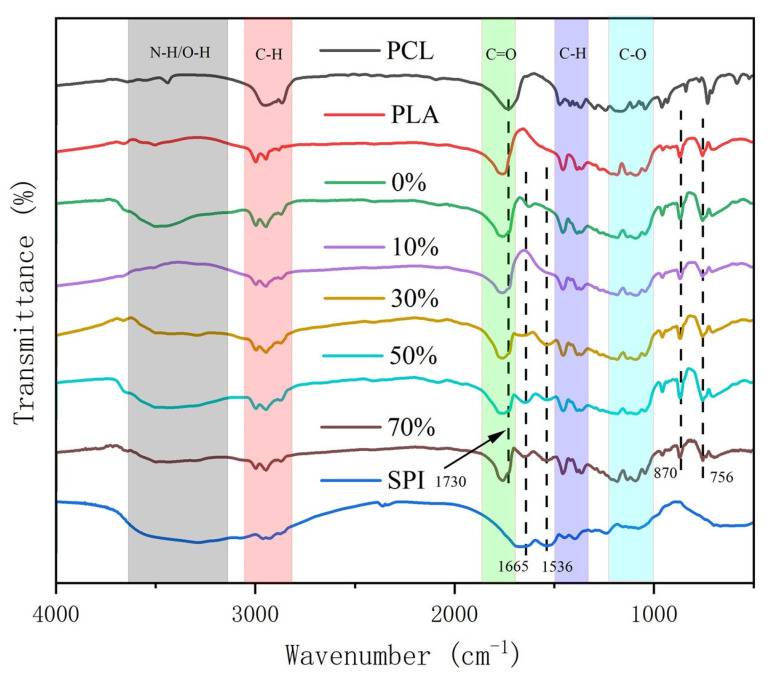
Infrared spectrum of nSPI/PLA–PCL composite microspheres.

Fourier transform infrared spectroscopy was used to detect the chemical composition and physical structure of the sample, and the FTIR spectra of nSPI/PLA–PCL composite porous microspheres were obtained. The analysis showed that 3502 cm^−1^ was the characteristic absorption peak of end–OH, and 2996 cm^−1^, 2946 cm^−1^ and 2881 cm^−1^ were the stretching vibration peaks of C–H in PLA. There is a C=O stretching vibration peak at 1759 cm^−1^, and 1044 cm^−1^ and 1185 cm^−1^ are C–O–C stretching vibrations, indicating the existence of ester groups. In PCL, 1367 cm^−1^ is the stretching vibration peak of C–H, 1166 and 1047 cm^−1^ are the stretching vibration peaks of ester C–O, 1730 cm^−1^ is the stretching vibration peak of C=O, and 1367 cm^−1^ is the bending vibration peak of C–H. The absorption peak of SPI at 3285 and 3074 cm^−1^ is the stretching vibration of hydroxyl O–H and amino N–H; the absorption peak at 1665 cm^−1^ is the stretching vibration of amide C=O; the absorption peak at 1536 cm^−1^ is the bending vibration of amide C–N–H; the absorption peak at 1238 cm^−1^ is the stretching vibration of amide C–N. The absorption peak at 1079 cm^−1^ is the stretching vibration of C–C, and the absorption peak at 668 cm^−1^ is the bending vibration of amide NH and O=C–N. As can be seen from Figure 3, no new combination bonds were formed in the composite microspheres, indicating that SPI and PLA/PCL were physically bound rather than chemically bound. In the composite microsphere experimental group, we can all see the characteristic peaks of the three substances, of which the curve changes in the 10% group are inconsistent with the other four groups, but the curve changes are consistent, which may be caused by some factors in the test. With the increase in SPI content, the absorption peaks of 30%, 50% and 70% groups at 1665 and 1536 cm^−1^ are the stretching vibration of C=O in the amide I band and the bending vibration of C–N–H in the amide II band. However, the two absorption peaks did not appear in the 10% group, which may be the reason for the low SPI content.

#### 3.1.4. XRD of nSPI/PLA–PCL Composite Microspheres

**Figure 4:**
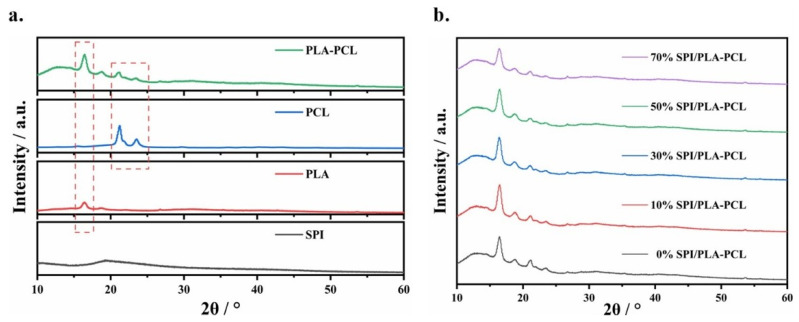
XRD patterns of nSPI/PLA–PCL composite microspheres. (**a**) Control groups; (**b**) Composite microspheres with different SPI contents.

Figure 4 shows the XRD pattern of nSPI/PLA–PCL composite microspheres after freeze–drying. As can be seen from Figure 4a, the peak value of PLA is slightly lower due to the small amount of PCL, and the addition of PCL increases the crystallinity of PLA slightly. nSPI/PLA–PCL composite microspheres showed characteristic peaks of each component in each group, and no new crystallization peaks appeared, indicating that nSPI/PLA–PCL composite microspheres were successfully synthesized (Figure 4b). This is consistent with the FTIR analysis results.

### 3.2. The Porosity of Composite Materials

The porosity of nSPI/PLA–PCL composite microspheres (*n* = 0%, 10%, 30%, 50%, 70%) in each group was 83.47%, 86.29%, 87.84%, 88.94%, 93.07%, respectively, and the porosity of composite microspheres was above 80%, and with the increase in SPI content, the porosity of NSPI composite microspheres was above 80%. The porosity increased gradually, and the porosity of the 70% group reached 93.07%, which met the porosity requirement of bone regeneration materials.

The average porosity of the five groups of composite materials was measured by the mercury injection method, and it was found that all the composite porous microspheres had good porosity, which was basically kept above 80%, which was the reason for the existence of pore–making agent NH_4_HCO_3_. The mechanism of NH_4_HCO_3_ pore–formation is as follows: in the process of emulsification into pellets, NH_4_HCO_3_ is decomposed into NH3 and CO_2_ under high–speed dispersion, and the two are released from the inside of the microsphere in the form of bubbles, thus leaving holes in the inside and surface of the microsphere, and the pore–formation effect is better. Therefore, in this paper, NH_4_HCO_3_ was selected as the pore–causing agent, the mass fraction of 1% was the most suitable, and the pore–causing effect was obvious. It is found that the porosity and the intercommunication within the pores are important factors for the type and amount of bone growth. When the porosity exceeds 30%, the pores can communicate with each other, and the new bone tissue can grow into the inside of the material through the surface pores [45]. When the porosity is greater than 90%, the material has a high specific surface area, which is conducive to the migration of cells, the transport of nutrients and the expulsion of metabolites [46]. On the premise of ensuring the mechanical properties of the material, the higher the porosity, the more conducive it is to the growth of bone tissue.

### 3.3. Water Contact Angle of Composite Microsphere

The interaction between biomaterials and cells mainly occurs on the surface of the material. Therefore, the surface properties of biomaterials and the response behavior of cells on the surface of the material have an important impact on the biocompatibility of the material [47]. The interaction between cells and biomaterials is a progressive dynamic process, which is divided into several stages: protein adsorption, cell contact, cell adhesion, and cell spreading [48]. For adherent growth cells (bone marrow mesenchymal stem cells, fibroblasts, etc.), after adhering to the surface of the material, they then guide the migration, proliferation and differentiation of subsequent cells through the joint action of biological molecules such as extracellular matrix proteins, cell membrane proteins and cytoskeleton proteins. The main properties that affect cell behavior are the chemical composition of materials, surface charge and surface hydrophilicity [49].

The surface properties of biomaterials are closely related to the biological properties of the material, and the hydrophilic/hydrophobic properties of the surface of the material are very important. Strong hydrophobic biomaterials are more likely to adsorb proteins to the surface, and hydrophilic biomaterials are more conducive to cell adhesion to the surface of the material [50]. After biomaterials are implanted in the body, proteins are first adsorbed on the surface of the biomaterials, and then regulate subsequent cell behavior. It has been reported that after planting a large number of cells on the surface of PLGA microspheres, the adhesion and proliferation of cells are not affected, but for hydrophobic PLGA porous microspheres, it is not easy for the cells to attach to the surface and enter the interior of the microspheres. Liu Yaoshan et al. [51] changed the hydrophobic PLGA surface into a hydrophilic surface through sodium hydroxide pretreatment and negative pressure operation, and fibroin modification at the same time, so that it was easy for the cells to attach to the surface of the microsphere and enter the scaffold.

**Figure 5:**
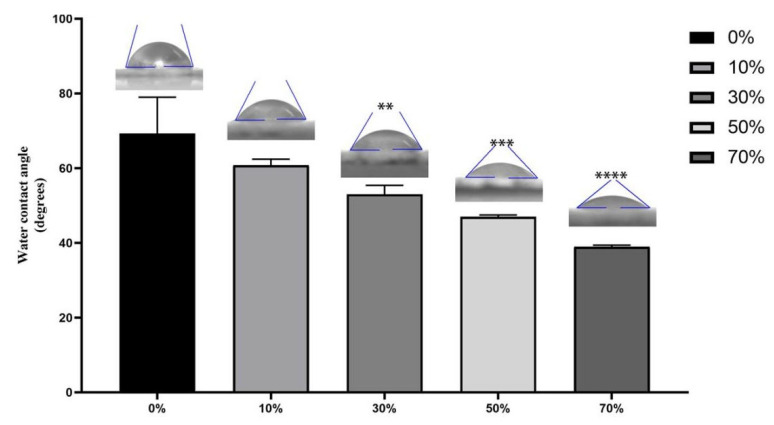
Water contact angle of nSPI/PLA–PCL composite microsphere, **: *p* < 0.01; ***: *p* < 0.001, ****: *p* < 0.0001, *n* = 3.

SPI has good hydrophilicity, with hydrophilic groups such as –NH2, –COO and –CONh distributed along the main chain of proteins [52], and there are strong hydrogen bonds, dipole interactions, electrostatic interactions, hydrophobic interactions and disulphide covalent bonds between SPI molecules and molecules [53]. These hydrophilic groups and chemical bonds can pull the water molecules, causing the water molecules to adsorb water around the protein molecules. Both PLA and PCL exhibit hydrophobicity, and the surface of hydrophobic materials is not conducive to cell adhesion and proliferation, so the hydrophobicity of composites can be improved by adding hydrophilic materials. Therefore, this research group added hydrophilic SPI to PLA/PCL. According to the test results of the water contact angle of microspheres in each group (see Figure 5), the addition of SPI changed the hydrophobicity of the PLA/PCL material. The water contact angle of the 0% SPI/PLA–PCL composite microsphere is 69.33 ± 9.68. The water contact angles of nSPI–PLA/PCL composite microspheres with different SPI contents (10%, 30%, 50%, 70%) were 60.81 ± 1.63, 53.06 ± 2.73, 47.03 ± 0.44, 38.97 ± 0.44, respectively. With the increase in SPI content, the water contact competition of composite microspheres gradually decreased, indicating that the hydrophilicity of composite microspheres was gradually enhanced, and the water contact angle of composite microspheres in the 30%, 50% and 70% groups was statistically significant compared with that in the 0% group. It can be seen that the addition of SPI can provide significant biological activity for composite microspheres.

In addition, the tertiary structure of SPI is composed of a hydrophobic intermediate region and an outer region composed of hydrophilic amino acid residues, and the interaction of hydrophobic groups is the most important force to maintain the tertiary structure of proteins. Wang S et al. [54] fabricated soy protein isolate/cellulose nanocrystal composite nanoparticles (SPI-CNC) via composite copolymerization strategy. Curcumin, which suffers from poor water solubility, easy oxidation in vitro and low oral absorption, was successfully encapsulated into the hybrid nanocarrier through mutual matrix assembly. This system effectively elevates local drug accumulation, optimizes therapeutic outcomes and alleviates potential systemic toxic side effects. Li, C. et al. [55] prepared SPI–modified reclaimed cellulose microspheres by the high–voltage electrostatic method, tested the water contact angle of reclaimed cellulose/soy protein isolate microspheres, and found that after the composite microspheres were made of hydrophilic reclaimed cellulose and SPI, the water contact angle increased and the hydrophilicity decreased. The enhanced surface hydrophobicity of composite microspheres indicates that the conformation of SPI changes. Therefore, the change in SPI conformation can be judged by the strength of surface hydrophobicity of composite microspheres. Zhang, L. et al. [56] developed Docetaxel nanosuspension (DTX–NS) coated with denatured soybean protein isolate (SPI) using the antisolvent precipitation–ultrasound method, which thermally induced denatured SPI, so that the hydrophobic residues changed from embedding in the protein to exposing on the protein surface, resulting in increased water solubility and enhanced adsorption on the hydrophobic surface. Denatured SPI is used as a stabilizer for nanosuspensions to improve the water solubility of DTX, thereby improving its intracellular delivery.

### 3.4. Degradation Performance and pH Value Determination of Composite Microspheres

**Figure 6:**
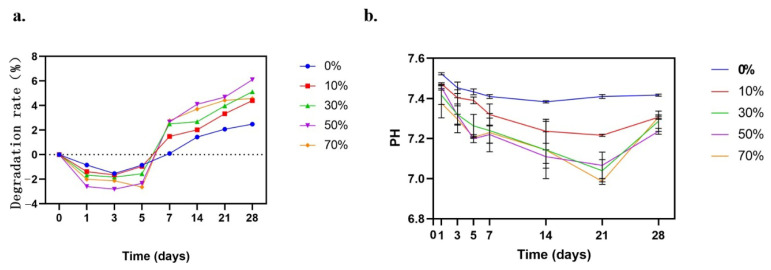
(**a**) Degradation rate curve of composite microspheres in PBS solution; (**b**) Change in pH value of composite microspheres in PBS solution.

Figure 6a shows the change process of mass loss rate of nSPI/PLA–PCL composite microspheres during the degradation cycle. With the increase in soaking time, the mass of composite microspheres decreased and the surface became rough, indicating that the microspheres were gradually degraded. It can be seen that the degradation trend of the five groups of samples is consistent, and the degradation of samples is mainly divided into two stages. In the first stage, about one week after degradation, the degradation rate of five groups of composite microspheres showed a negative change, which was the stage of sample weight gain. This is because the calcium phosphate in the solution is rapidly deposited on the surface of the composite microspheres and inside the pores, and gradually generates hydroxyapatite. In this process, the composite microspheres degrade simultaneously, but the degradation rate is lower than the deposition rate of calcium and phosphorus salt, so the overall performance is weight gain. In the second stage, after one week, the weight loss rate of the sample began to show a positive change. At this time, the sample itself began to degrade, and the degradation rate of the material was greater than the deposition rate of calcium and phosphorus salt in the solution, so the overall performance was weight loss. It can be seen from the degradation trend of the graph that the degradation of microspheres is relatively slow.

Figure 6b shows the PH change process of nSPI/PLA–PCL composite microspheres during the degradation cycle. As can be seen from the figure, the overall PH change presents a change of rising → decreasing → rising. On day 28, the PH of the 0% group reached about 7.42, 10% group reached about 7.31, 30% group reached about 7.29, 50% group reached about 7.23, and 70% group reached about 7.31. Overall, in the process of degradation, although the pH is reduced, it is always greater than 7, and theoretically it will not affect the surrounding tissues due to low pH, causing inflammation.

At present, the degradation mechanism of polylactic acid is still unclear, and it is generally believed that its degradation mainly starts from chemical hydrolysis, and the acid produced in the process may have a catalytic effect on the degradation, forming an autocatalytic effect [57]. The degradation rate of polylactic acid is mainly determined by the relative molecular weight, phase structure and morphology, and can be accelerated by adding some hydrophilic compounds or reducing the crystallinity. As shown in Figure 6, the degradation rate of 0% SPI/PLA–PCL composite porous microspheres without SPI was about 2% at 28 days, and the degradation rate of the other four groups was higher than that of the 0% group, among which the degradation rate of 50% SPI/PLA–PCL composite porous microspheres was the highest, about 6%. It can be seen that the addition of SPI has a certain effect on the degradation rate of composite microspheres. However, in the whole process, the degradation rate of nSPI/PLA–PCL composite porous microspheres was relatively slow, which may be due to the slow degradation rate of PLA itself, and the addition of PCL increased the crystallinity of PLA, resulting in the addition of SPI accelerating the degradation rate of nSPI/PLA–PCL composite porous microspheres.

### 3.5. In Vitro Mineralization of Composite Microspheres

**Figure 7:**
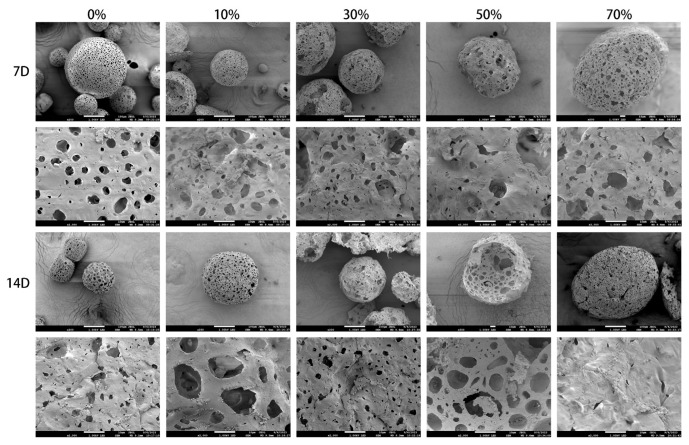
SEM images of nSPI/PLA–PCL composite microspheres soaked in SBF for 7 and 14 days.

As shown in Figure 7, the SEM map after incubation in simulated body fluid (SBF) for 7 and 14 days showed that short rod–like crystals appeared on the surface and inside the pores of the five groups of composite microspheres, and the crystals gradually increased with the increase in SPI content (*n* = 0%, 10%, 30%, 50%). The short rod crystals in the 70% group were less than those in other groups due to excessive SPI content. The composite microspheres degrade simultaneously in SBF, so it can be seen that the microspheres are spherical or quasi–spherical, the surface becomes rough, and the pores are more irregular. The mineralization and degradation were more obvious at 14 days than at 7 days.

**Figure 8:**
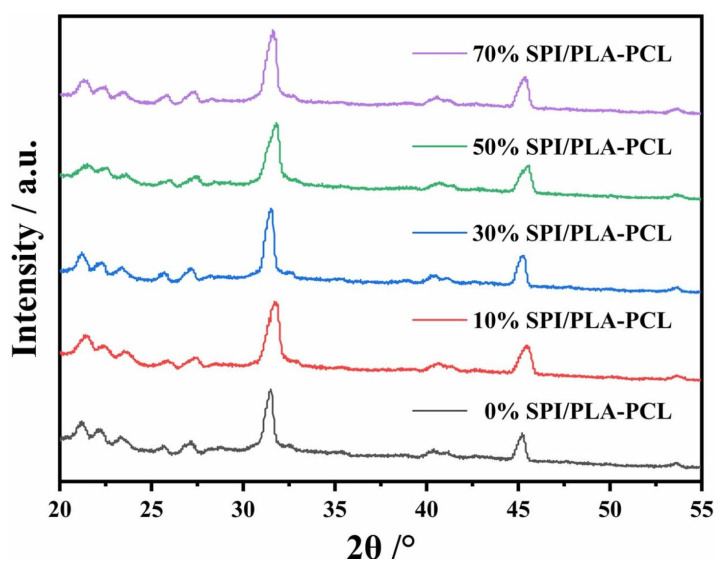
XRD pattern of nSPI/PLA–PCL composite microspheres soaked in SBF for 14 days.

As shown in Figure 8, XRD patterns after 14 days of incubation in simulated body fluid (SBF) showed characteristic apatite peaks corresponding to 26.0° (002) and 31.77° (211) (JCPDS9432 CAL), confirming hydroxyl apatite (HA) nucleation in all samples. Moreover, with the increase in SPI content (*n* = 0%, 10%, 30%, 50%), the characteristic peaks of apatite gradually become stronger, indicating that the addition of SPI can improve the mineralization ability of composite materials.

### 3.6. Composite Microsphere Differential Thermal Analysis

**Figure 9:**
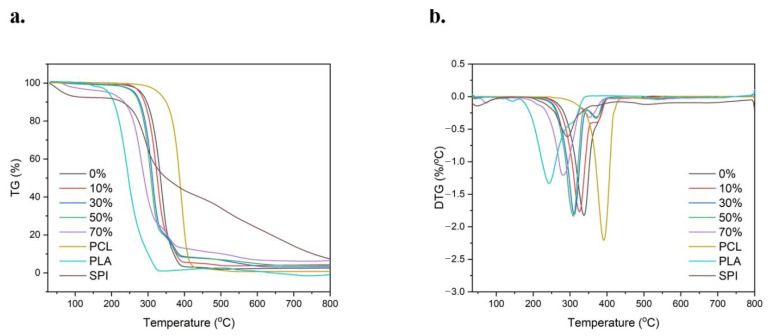
TG (**a**) and DTG (**b**) spectra of nSPI/PLA–PCL composite microspheres.

Figure 9 shows the TG and DTG curves of composite microspheres. It can be seen from the figure that the thermal decomposition of nSPI/PLA–PCL composite porous microspheres is roughly divided into three stages. In the first stage, from room temperature to about 200 °C, the microspheres have only a small amount of weight loss, which is caused by the evaporation of a small amount of bound water in the sample, and the microspheres hardly decompose. nSPI/PLA–PCL composite porous microspheres can be sterilized under high pressure at 121 °C, which meets the conventional sterilization requirements when used as biological materials. In the second stage, from 200 °C to 400 °C, all the samples showed a large weight loss step, which was caused by the C–chain decomposition of PLA/PCL and the decomposition reaction of SPI molecules at high temperature. The third stage is from 400 °C to 800 °C, so the weight of the sample shows only a slight change. As the SPI content increases from 0% to 50%, the maximum thermal decomposition temperature (the temperature at the time of maximum mass loss) of the microspheres decreases from 337 °C to 308 °C. In general, the thermal stability of composite porous microspheres with different contents of nSPI/PLA–PCL (*n* = 0%, 10%, 30%, 50%) is similar, and all show good thermal stability.

### 3.7. Hemolysis Test

Blood compatibility is the interaction between the surface of a biomaterial and various components of blood. When biological materials come into contact with blood, they will cause a series of defense reactions such as protein adhesion, platelet aggregation, thrombosis, activation of coagulation and complement systems, and hemolysis. Therefore, the surface properties of biological materials determine the biological reactions between materials and blood and tissues, which requires that biological materials need to have good blood compatibility.

Good blood compatibility requires not only good anticoagulant properties, but also good anti–hemolysis properties. Hemolysis is a common adverse reaction when biological materials come into contact with blood. When the two come into contact, hemolysis occurs due to the rupture of red blood cells in the blood and the release of intracellular hemoglobin into the plasma [58]. The in vitro hemolysis test is a simple and effective method to evaluate the anti–hemolysis performance of biomaterials. Hemolysis rate is assessed by measuring the relative amount of hemoglobin released into solution by red blood cells in whole blood exposed to the experimental material [59]. It is generally believed that when the HR value measured in the experiment is less than 5%, the material will not cause hemolysis, and the smaller the HR value measured, the smaller the impact and damage of the material on red blood cells.

**Figure 10:**
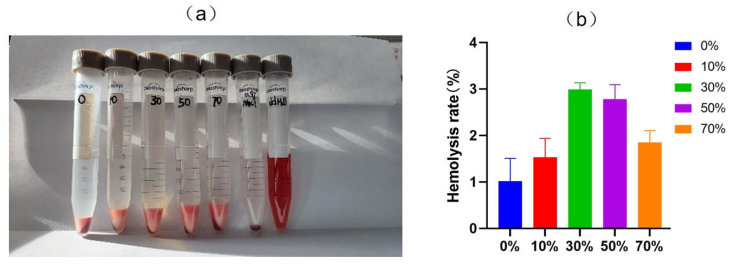
Hemolysis rate of nSPI/PLA–PCL composite microspheres. (**a**) Appearance. (**b**) Column diagram of hemolysis rate.

After implantation of nSPI/PLA–PCL composite microspheres as bone regeneration material, the material will come into contact with human blood, so it is necessary to investigate the blood compatibility of the composite material. As shown in Figure 10, it can be seen from Figure 10a that after centrifugation, stratification occurred in the experimental group and the negative control group, while hemolysis occurred in the positive control group. As can be seen in Figure 10b, the hemolysis rate of microspheres in all groups was below 3%, which met the relevant requirements of the standard hemolysis test (<5%) in ISO10993–4:22 Biological Evaluation of Medical Devices Part 4: Selection of tests for interaction with Blood [60]. The results showed that NSPI–PLA/PCL composite microspheres with different SPI contents (0%, 10%, 30%, 50%, 70%) did not cause obvious hemolysis, and it could be considered that nSPI/PLA–PCL composite microspheres had low/no toxicity to red blood cells. After implantation, the surface composite microspheres will not cause the rupture of red blood cells due to contact with blood, avoiding the possibility of cytotoxicity caused by the release of hemoglobin.

After the rupture of red blood cells, not only does hemolysis occur, but also some small molecules such as ADP may be released, which can cause the adhesion, aggregation and activation of platelets, resulting in a series of consequences such as coagulation. Therefore, related experiments such as a platelet agglutination test should be added in the future to improve the blood compatibility evaluation of nSPI/PLA–PCL composite microspheres.

### 3.8. Cell Test Results

#### 3.8.1. Isolation and Culture of MC3T3–E1 Cells and Proliferation and Viability of CCK8 Cells

**Figure 11:**
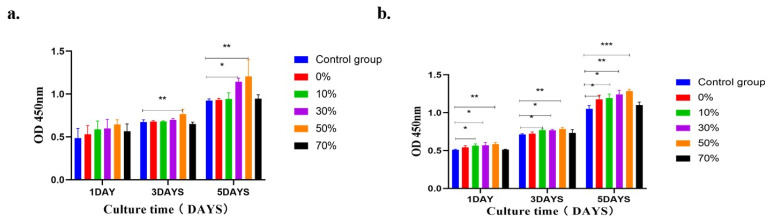
The effects of different concentrations of nSPI/PLA–PCL composite microspheres on the proliferation of MC3T3–E1 cells were detected by CCK–8, and (**a**) 100% extract, (**b**) 50% extract. *: *p* < 0.05; **: *p* < 0.01; ***: *p* < 0.001, *n* = 3.

**Figure 12:**
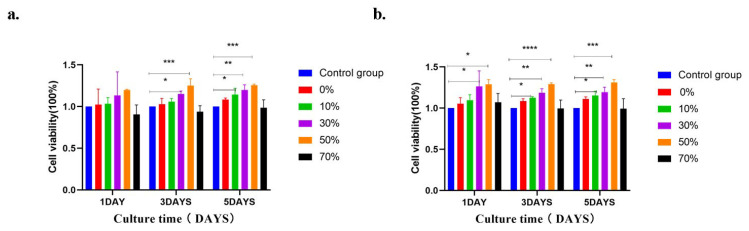
The effects of different concentrations of composite microspheres on the proliferation rate of MC3T3–E1 cells were as follows: (**a**) 100% extract, (**b**) 50% extract. *: *p* < 0.05; **: *p* < 0.01; ***: *p* < 0.001; ****: *p* < 0.0001, *n* = 3.

As shown in Figure 11, the effect of different groups of microsphere extracts on MC3T3–E1 cell proliferation is shown. In order to dynamically monitor the long–term proliferation trend of cells, CCK–8 quantitative detection is performed at three time points on days 1, 3 and 5 of this experiment. MC3T3 –E1 cells are cultured in nSPI/PLA–PCL composite microsphere extract, and the cells grow well. Under the culture condition of 100% microsphere extract (Figure 11a), there was no statistical difference between cell proliferation and the negative control group at the same time on day 1, and there was a statistical difference between the 50% group on day 3 and the 30% and 50% groups on day 5. The 10%, 30%, 50% concentrations of composite microsphere extract compared to the negative control at the same time (Figure 12b). Groups were statistically different at each time point. The results showed that the microspheres had good biocompatibility and promoted cell adhesion and proliferation.

Figure 12 shows the relative proliferation rate of MC3T3–E1 cells cultured with 100% and 50% microsphere extracts. Compared with the negative control group at the same time, the MC3T3–E1 cells in the five experimental groups were able to grow and multiply well and did not show obvious cytotoxicity. In the 100% concentration extract (Figure 12a), compared with the negative control group, there was no statistical significance in the cell proliferation rate on the 1st day, while there was statistical significance in the 30% and 50% groups on the 3rd and 5th day. In the 50% concentration extract (Figure 12b), compared with the negative control group, the cell proliferation rate in the 30% and 50% groups was statistically significant on the 1st, 3rd, and 5th days, while that in the 10% group was statistically significant on the 3rd and 5th days. According to the ISO10993–5 standard [61] (Table 3), the cytotoxicity of the composite extracts of the five experimental groups was 0–1, indicating that nSPI/PLA–PCL composite microspheres had good biocompatibility.

The results of CCK8 cell proliferation showed that the addition of soy protein isolate improved the cellular activity of nSPI/PLA–PCL composite porous microspheres. With the increase in time, an appropriate amount of soy protein isolate was gradually hydrolyzed, and some amino acid nutrients were released in the cell medium to promote cell growth. However, an SPI concentration too high (70% nSPI/PLA–PCL composite microspheres extract) could inhibit cell growth, which may be due to excessive activation or amino acids destroying the nutrient balance and osmotic pressure homeostasis of the cell culture medium [62], over–activating the mTOR proliferation pathway, inducing oxidative stress and apoptosis of cells [63,64]. In addition, high SPI content could also change the physicochemical properties of microspheres, which could further aggravate the cell growth inhibition effect [65]. In general, the proliferation activity of MC3T3–E1 cells in all groups with nSPI/PLA–PCL composite microspheres (*n* = 0%, 10%, 30%, 50%) was higher than that in the control group (pure DMEM medium) at every time point. These results indicated that these composite microspheres had no obvious toxic effects on cells, and even promoted the proliferation of MC3T3–E1 cells, indicating that the composite microspheres had good cytocompatibility, which was consistent with the results of the CCK8 cytotoxicity test.

#### 3.8.2. Cell Adhesion and Growth on Microspheres

**Figure 13:**
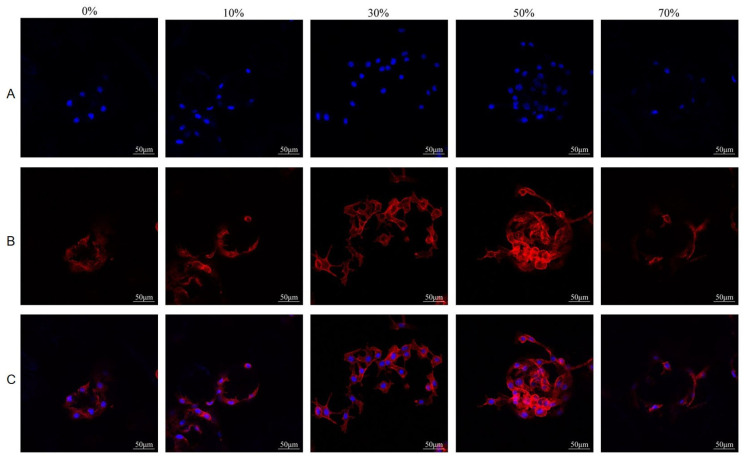
Laser confocal results of MC3T3–E1 cells cultured with nSPI–PLA/PCL composite microspheres for 72 h ((**A**) DAPI, (**B**) Rhodamine Phalloidin, (**C**) Merge).

Figure 13 shows the laser confocal observation results of microsphere co–culture, and the surface profile, cell morphology and adhesion of the material can be observed. The cell morphology of all groups was good, and the cells spread on the surface of the composite microsphere combined with the surface of the microsphere and covered the surface of the microsphere. The adhesion and proliferation of the microsphere cells in all groups were similar, indicating that the nSPI/PLA–PCL composite microsphere had good biocompatibility.

**Figure 14:**
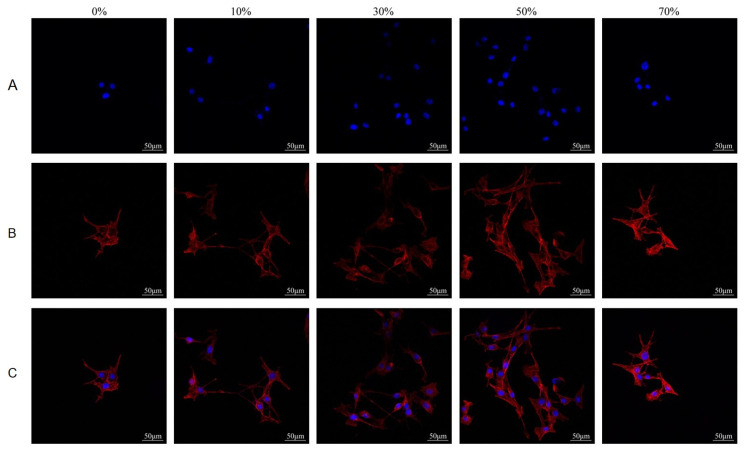
Laser confocal observation of MC3T3–E1 cells co–cultured with nSPI–PLA/PCL composite microsphere extract for 72 h ((**A**) DAPI, (**B**) Rhodamine Phalloidin, (**C**) Merge).

Laser confocal observation results of cell morphology after culture of microsphere extract are shown in Figure 14. Cell adhesion and pseudopodia extension can be observed. MC3T3–E1 cells were cultured with different microsphere extracts for 72 h, and the growth of the cells was good; the cells did not have obvious morphological changes, the cells extended a lot of filopodia, and the surrounding cells adhered well. There was no significant difference in the morphology of the cells cultured by microsphere extract in each group, and the cell adhesion and proliferation were similar. These results indicate that the five groups of composite microspheres have good biocompatibility and can promote the early proliferation and adhesion of cells.

For bone tissue engineering materials, many factors affect cell adhesion, migration and proliferation. These factors mainly include material composition, surface roughness, pore structure and particle size. The contours of the material and the adhesion and extension of the cells can be clearly seen from the confocal images. The cells can adhere to the surface of the microsphere well and spread out along the surface of the microsphere with many filamentous pseudopods, there is no significant difference in cell morphology among the groups, and the cell number is similar to the proliferation result, especially the 50% SPI/PLA–PCL composite microsphere group. The cells adhered and grew almost uniformly on the entire surface of the microsphere, and the cell density on the surface was higher than in the other groups. The results showed that 50% SPI/PLA–PCL composite microspheres had the best potential for bone tissue engineering and were expected to be used as bone tissue engineering materials.

#### 3.8.3. Alkaline Phosphatase ALP

**Figure 15:**
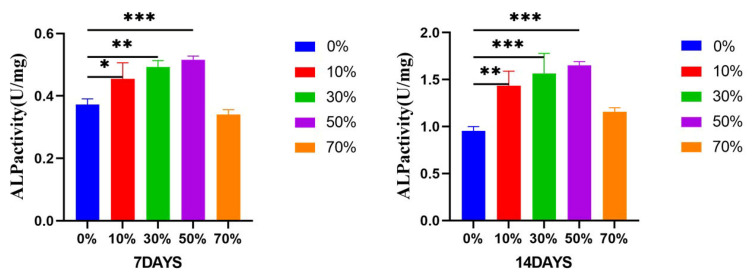
Effect of 50% extract of nSPI/PLA–PCL composite microspheres on the expression of ALP in MC3T3–E1 cells, *: *p* < 0.05; **: *p* < 0.01; ***: *p* < 0.001, *n* = 3.

Figure 15 shows the expression of alkaline phosphatase at day 7 and day 14 after co–culture of 50% extracts of different composite microspheres with MC3T3–E1 cells. The effect of composite microspheres on the differentiation of MC3T3–E1 cells could be reflected by detecting the level of alkaline phosphatase at different time points. The levels of alkaline phosphatase in the 10%, 30% and 50% groups were statistically different at day 7 and day 14, and 50% were higher than the 10% and 30% groups. There was no significant difference in the 70% group.

Alkaline phosphatase is one of the signature enzymes in the early differentiation of osteoblasts [66] and plays a key role in osteogenesis [67]. The main function of alkaline phosphatase is to hydrolyze phosphate ester and pyrophosphate in the process of osteogenesis, provide phosphoric acid for the deposition of hydroxyapatite, and relieve the inhibition of pyrophosphate in the process of bone salt formation, which is conducive to bone formation [68].

However, the increase in local phosphoric acid content can promote matrix mineralization and calcium salt deposition in osteoblasts, which is a specific index to evaluate the osteogenic activity and tissue calcification ability of the body [69]. Since the expression activity of alkaline phosphatase is an obvious feature of osteoblast differentiation, its expression activity increases with the increase in cell differentiation in the process of osteogenesis [70]. Figure 15’s ALP results showed that with the increase in SPI content in composite microspheres, the activity of alkaline phosphatase secreted by cells increased with a statistically significant difference. ALP expression: group 50% > group 30% > Group 10% > group 0%, which may be because 50% SPI/PLA–PCL composite microspheres have the most significant adhesion and proliferation effect on MC3T3–E1 cells. Compared with other groups with weak adhesion and proliferation effect, a large number of MC3T3–E1 cells adhere to the surface of 50% SPI/PLA–PCL composite microspheres. In addition, MC3T3–E1 cells were more conducive to oriented differentiation into osteoblasts and increased ALP expression activity under the influence of mineralized induction medium.

#### 3.8.4. Alizarin Red Staining

**Figure 16:**
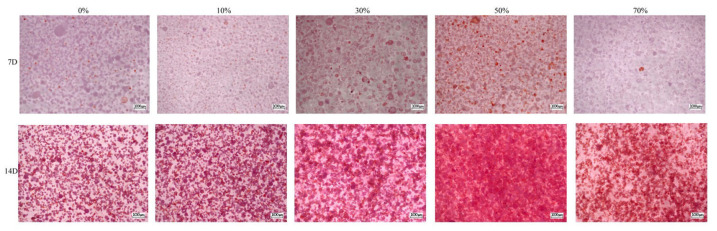
nSPI/PLA–PCL composite microspheres and MC3T3–E1 cells were co–cultured in mineralized induction medium for 7 days and 14 days with alizarin red staining.

**Figure 17:**
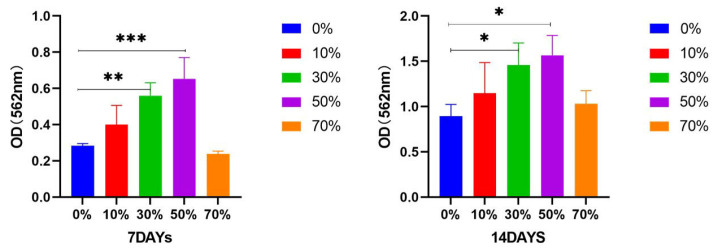
Semi–quantitative analysis of alizarin red staining of nSPI/PLA–PCL composite microspheres and MC3T3–E1 cells cultured in mineralized induction medium for 7 and 14 days, *: *p* < 0.05; **: *p* < 0.01; ***: *p* < 0.001, *n* = 3.

Alizarin red staining is a common cell staining method that can be chelated with calcium salts to form orange–red complexes and is commonly used to assess mineral deposits in osteoblasts [71]. The content of cellular calcium deposition is a late marker of extracellular matrix maturation, and its content indirectly reflects the degree of differentiation and mineralization of MC3T3E1 cells into osteoblasts [72]. As shown in Figure 16, after different composite microspheres were cultured with MC3T3–E1 cells, alizarin red staining was obtained at 7 and 14 days after mineralization induction. Spherical particles were composite microspheres, and purpure–orange–red staining was mineralized nodules. It can be seen that the staining of nSPI/PLA–PCL composite microspheres (*n* = 0%, 10%, 30%, 50%) gradually deepened with the increase in SPI content, and the orange coloring gradually increased, indicating that the mineralization nodules gradually increased, indicating that the mineralization ability was gradually enhanced. Further, 70% SPI/PLA–PCL composite microspheres, due to excessive SPI content, are not conducive to cell adhesion and proliferation, so the orange color is reduced, and the mineralization ability is weakened. The effect of 14 days was more obvious than that of 7 days. Figure 17’s semi–quantitative analysis results show that the mineralized water level of the 30% and 50% groups at day 7 and day 14 is significantly higher than that of the 0% group at the same time period, while there is no significant difference in the mineralized water level of the 10% group and 70% group. These results indicate that SPI has the ability to promote calcium deposition during the formation of extracellular matrix (ECM).

## 4. Conclusions

1. nSPI/PLA–PCL composite microspheres were successfully prepared by the multi–emulsion solvent volatilization method, the porosity of composite microspheres in all groups was higher than 80%, and the porosity of 70% SPI/PLA–PCL composite microspheres was 93.07%, which met the requirements of bone tissue engineering.
2. The addition of SPI improves the bioactivity, degradation rate, biocompatibility and osteogenic inductivity of nSPI/PLA–PCL composite microspheres. Further, 50% SPI/PLA–PCL composite microspheres showed obvious advantages over other groups in cell proliferation, adhesion, ALP and alizarin red staining, and had potential application in tissue engineering.

## Figures and Tables

**Table 1:** Main reagents.

Raw Material	Molecular Formula/Model	Manufacturer
Polylactic acid	(C_3_H_4_O_2_)n	Shanghai Macklin Biochemical Technology Co., Ltd., Shanghai, China
Polycaprolactone	(C_6_H_10_O_2_)n	Shanghai Macklin Biochemical Technology Co., Ltd.
Soy protein isolate	C_13_H_10_N_2_	Shanghai Macklin Biochemical Technology Co., Ltd.
Ammonium bicarbonate	NH_4_HCO_3_	Sinopharm Chemical Reagents Co., Ltd., Beijing, China
Polyvinyl alcohol	(C_2_H_4_O)n	Sinopharm Chemical Reagents Co., Ltd.
triple distilled water	H_2_O	Provided by the Life Science Experiment Center of Jiamusi University, Jiamusi, Heilongjiang, China
DMEM medium	C0891–500 mL	Shanghai Beyotime Biotechnology Co., Ltd., Shanghai, China
Fetal bovine serum	04–001–1A	BI (Biolnd), Kibbutz Beit Haemek, Israel
Penicillin–streptomycin biantibodies	C0222	Shanghai Beyotime Biotechnology Co., Ltd.
0.25% trypsin digest	C0201–100 mL	Shanghai Beyotime Biotechnology Co., Ltd.
Alizarin red kit	C0140–100 mL	Shanghai Beyotime Biotechnology Co., Ltd.
Alkaline phosphatase kit	P0321M	Shanghai Beyotime Biotechnology Co., Ltd.
CCK8 kit		Aperbio Biotech (Suzhou) Technology Co., Ltd., Suzhou, Jiangsu, China
MC3T3–E1 cells		Provided by the team of Professor Zhang Huiming of Basic Medicine of Jiamusi University, Jiamusi, Heilongjiang, China
Simulate body fluids	G0390–500 mL	Solarbios Science & Technology Co., Ltd., Beijing, China

**Table 2:** Instruments and equipment.

Name	Model	Manufacturer
Electronic balance	FA2004	Shanghai Shunyu Hengping Scientific Instrument Co., Ltd., Shanghai, China
Magnetic stirrer	DF–101S	Gongyi Yuhua Instrument Co., Ltd., Gongyi, China
High–speed disperser	FS–680	Yaku Electromechanical
X–ray diffractometer rays	BRUKER D8 ADVANCE	BRUKER Corporation, Billerica, MA, USA
Scanning electron microscope	TESCAN MIRA LMS	TESCAN Brno, s.r.o., Brno, Czech Republic
Fourier transform infrared spectrometer	Thermo Scientific Nicolet iS20	Thermo Fisher Scientific, Waltham, MA, USA
Thermogravimetric analyzer	TA Discovery TGA 550	TA Instruments, New Castle, DE, USA
Centrifuge	TG16G	Henan Beihong Industrial Co., Ltd., Zhengzhou, China
Freeze dryer	Vir Tis Wizard 2.0	SP Scientific, Warminster, PA, USA
Constant temperature water bath	HH–US–B	Jincheng Chunlan Experimental Instrument Factory, Jintan District, China
Fully automatic multifunctional microplate reader	BioTek Synergy HT	BioTek Instruments, Inc., Winooski, VT, USA
Electric constant temperature incubator	DHP–420	Changzhou Zhongjie Experimental Instrument Manufacturing Co., Ltd., Changzhou, China
Contact angle measuring instrument		Zhongchen Digital Technology Equipment Co., Ltd., Jintan District, China

**Table 3:** The relationship between cell proliferation rate and cytotoxic reaction grade.

Rank	Relative Growth Rate
0	≥100
1	80–99
2	50–79
3	30–49
4	0–29

## Data Availability

The data is contained within the article.

## Abbreviations

The following abbreviations are used in this manuscript:

EDS	Energy Dispersive Spectrometer
XRD	X–ray diffraction
SEM	Scanning Electron Microscope
FTIR	Fourier Transform Infrared Spectrometer
SPI	Soy protein isolate
PLA	Polylactide
PCL	Polycaprolactone
ALP	Alkaline phosphatase
ECM	extracellular matrix
FBS	fetal bovine serum
SBF	simulated body fluid
PBS	Phosphate–Buffered Saline
OD	Optical Density

## References

[1] Jimi E., Hirata S., Osawa K., Terashita M., Kitamura C., Fukushima H. (2012). The current and future therapies of bone regeneration to repair bone defects. Int. J. Dent..

[2] Cao G.D., Pei Y.Q., Liu J., Li P., Liu P., Li X.S. (2021). Research progress on bone defect repair materials. Zhongguo Gu Shang.

[3] Qu H., Fu H., Han Z., Sun Y. (2019). Biomaterials for bone tissue engineering scaffolds: A review. RSC Adv..

[4] Fraile-Martínez O., García-Montero C., Coca A., Álvarez-Mon M.A., Monserrat J., Gómez-Lahoz A.M., Coca S., Álvarez-Mon M., Acero J., Buján J. (2021). Applications of polymeric composites in bone tissue engineering and jawbone regeneration. Polymers.

[5] Guo L., Liang Z., Yang L., Du W., Yu T., Tang H., Li C., Qiu H. (2021). The role of natural polymers in bone tissue engineering. J. Control. Release.

[6] Putra N.E., Zhou J., Zadpoor A.A. (2024). Sustainable sources of raw materials for additive manufacturing of bone-substituting biomaterials. Adv. Healthc. Mater..

[7] Dimitriou R., Jones E., McGonagle D., Giannoudis P.V. (2011). Bone regeneration: Current concepts and future directions. BMC Med..

[8] Chen L., Ramezan Y., Pourramezan H., Najafi A., Kamkari A., Goksen G., Huang Z., Zhang W. (2025). Soy protein isolate (SPI)-based films/coatings for food packaging: Research progress on properties and applications. Compr. Rev. Food Sci. Food Saf..

[9] Zhao W., Yang X., Li L. (2024). Soy protein-based wound dressings: A review of their preparation, properties, and perspectives. ACS Appl. Mater. Interfaces.

[10] Lu J., Xu Q., Ma J., Xu X., Zhao Y., Pu Y., Deng Y., Yan K., Zong Y., Fan Q. (2025). Recent progress in protein-based food packaging films. Collagen Leather.

[11] Ahn S., Chantre C.O., Gannon A.R., Lind J.U., Campbell P.H., Grevesse T., O’Connor B.B., Parker K.K. (2018). Soy protein/cellulose nanofiber scaffolds mimicking skin extracellular matrix for enhanced wound healing. Adv. Healthc. Mater..

[12] Huo J., Wang L., Su S., Yu X., Duan Y., Wang P., Xiao Z. (2025). Development and characterization of soy protein isolate-based emulsion films with green sichuan pepper essential oil: Functional and structural insights. Food Res. Int..

[13] Chen M., Yuan Q., Lin Z. (2024). Development and characterization of soy protein isolate/xylose film. Int. J. Polym. Sci..

[14] Yang Z., Yin G., Sun S., Xu P. (2024). Medical applications and prospects of polylactic acid materials. iScience.

[15] Khouri N.G., Bahú J.O., Blanco-Llamero C., Severino P., Concha V.O.C., Souto E.B. (2024). Polylactic acid (PLA): Properties, synthesis, and biomedical applications—A review of the literature. J. Mol. Struct..

[16] Castañeda-Rodríguez S., González-Torres M., Ribas-Aparicio R.M., Del Prado-Audelo M.L., Leyva-Gómez G., Gürer E.S., Sharifi-Rad J. (2023). Recent advances in modified poly(lactic acid) as tissue engineering materials. J. Biol. Eng..

[17] Zarei M., Sayedain S.S., Askarinya A., Sabbaghi M., Alizadeh R. (2023). Improving physio-mechanical and biological properties of 3D-printed PLA scaffolds via in-situ argon cold plasma treatment. Sci. Rep..

[18] Momeni S., Craplewe K., Safder M., Luz S.M., Sauvageau D., Elias A. (2023). Accelerating the biodegradation of poly(lactic acid) through the inclusion of plant fibers: A review of recent advances. ACS Sustain. Chem. Eng..

[19] Mat Piah M.B., Ahmad M.N., Abdullah E.N., Muzakkar M.Z. (2023). Modifications of poly(lactic acid) with blends and plasticization for tenacity and toughness improvement. Indones. J. Chem..

[20] Feng P., Jia J., Liu M., Peng S., Zhao Z., Shuai C. (2021). Degradation mechanisms and acceleration strategies of poly(lactic acid) scaffold for bone regeneration. Mater. Des..

[21] Maduka C.V., Alhaj M., Ural E., Habeeb O.M., Kuhnert M.M., Smith K., Makela A.V., Pope H., Chen S., Hix J.M. (2023). Polylactide degradation activates immune cells by metabolic reprogramming. Adv. Sci..

[22] Wu D., Samanta A., Srivastava R.K., Hakkarainen M. (2015). Substrate-anchored and degradation-sensitive anti-inflammatory coatings for implant materials. Sci. Rep..

[23] Fredericks C.M., Kunihiro J.K.I., Zheng H., Waghu N.R., Kamkar M. (2024). Chemical enhancements and advanced manufacturing methods of poly(lactic acid) for tissue engineering applications. Polymer.

[24] Wang W., Zhang B., Li M., Li J., Zhang C., Han Y., Wang L., Wang K., Zhou C., Liu L. (2021). 3D printing of PLA/n-HA composite scaffolds with customized mechanical properties and biological functions for bone tissue engineering. Compos. Part B Eng..

[25] Ramírez-Ruiz F., Núñez-Tapia I., Piña-Barba M.C., Alvarez-Pérez M.A., Guarino V., Serrano-Bello J. (2025). Polycaprolactone for hard tissue regeneration: Scaffold design and in vivo implications. Bioengineering.

[26] Ntrivala M.A., Pitsavas A.C., Lazaridou K., Baziakou Z., Karavasili D., Papadimitriou M., Ntagkopoulou C., Balla E., Bikiaris D.N. (2025). Polycaprolactone (PCL): The biodegradable polyester shaping the future of materials—A review on synthesis, properties, biodegradation, applications and future perspectives. Eur. Polym. J..

[27] Fortelny I., Ujcic A., Fambri L., Slouf M. (2019). Phase structure, compatibility, and toughness of PLA/PCL blends: A review. Front. Mater..

[28] Negaresh M., Javadi A., Garmabi H. (2024). Poly(lactic acid)/poly(ε-caprolactone) blends: The effect of nanocalcium carbonate and glycidyl methacrylate on interfacial characteristics. Front. Mater..

[29] Åkerlund E., Diez-Escudero A., Grzeszczak A., Persson C. (2022). The effect of PCL addition on 3D-printable PLA/HA composite filaments for the treatment of bone defects. Polymers.

[30] Fernández-Tena A., Guerrica-Echevarría G., Aranburu N., Wang Z., Cavallo D., Müller A.J. (2024). How to achieve a highly toughened 70/30 PLA/PCL blend by using nucleating agents and tailoring processing conditions. ACS Appl. Polym. Mater..

[31] Yang S., Wu H., Peng C., He J., Pu Z., Lin Z., Wang J., Hu Y., Su Q., Zhou B. (2024). From the microspheres to scaffolds: Advances in polymer microsphere scaffolds for bone regeneration applications. Biomater. Transl..

[32] Desai N., Pande S., Vora L.K., Kommineni N. (2024). Nanofibrous microspheres: A biomimetic platform for bone tissue regeneration. ACS Appl. Bio Mater..

[33] Iqbal M., Zafar N., Fessi H., Elaissari A. (2015). Double emulsion solvent evaporation techniques used for drug encapsulation. Int. J. Pharm..

[34] Li X., Li L., Wang D., Zhang J., Yi K., Su Y., Luo J., Deng X., Deng F. (2024). Fabrication of polymeric microspheres for biomedical applications. Mater. Horiz..

[35] Liu Y., Wu H., Jia Z., Du B., Liu D., Zhou Z. (2019). Silk fibroin-modified polylactic acid-glycolic acid copolymer porous microspheres as gingival mesenchymal stem cells delivery carrier. Chem. J. Chin. Univ..

[36] (2017). Implants for Surgery—Homopolymers, Copolymers and Blends on Poly(Lactide)—In Vitro Degradation Testing.

[37] (2021). Biological Evaluation of Medical Devices—Part 12: Sample Preparation and Reference Materials.

[38] Yang Y.Y., Chung T.S., Ng N.P. (2001). Morphology, drug distribution, and in vitro release profiles of biodegradable polymeric microspheres prepared by double-emulsion solvent extraction/evaporation method. Biomaterials.

[39] Cao Q., Chen J., Zhang Z., Xiong Y., Ma J., Sun W., Chen X., Lou Q., Tang K., Lin F. (2025). Faster efficacy and reduced nodule occurrence with PLLA (poly-L-lactic acid) porous microspheres. Front. Bioeng. Biotechnol..

[40] Yenying A., Tangamatakul K., Supanchart C., Jenvoraphot T., Manokruang K., Worajittiphon P., Punyodom W., Daranarong D. (2022). Preparation and characterization of PLG microparticles by the multiple emulsion method for the sustained release of proteins. Micromachines.

[41] Rosca I.D., Watari F., Uo M. (2004). Microparticle formation and its mechanism in single and double emulsion solvent evaporation. J. Control. Release.

[42] Cai Z., Jiang H., Lin T., Wang C., Ma J., Gao R., Jiang Y., Zhou X. (2022). Microspheres in bone regeneration: Fabrication, properties and applications. Mater. Today Adv..

[43] O’Flynn T.D., Hogan S.A., Daly D.F.M., O’Mahony J.A., McCarthy N.A. (2021). Rheological and solubility properties of soy protein isolate. Molecules.

[44] Monroy-Rodríguez I., López-Hernández R.E., Villalobos-Espinosa J.D.C., Jaimez Ordaz J., Cornejo Mazón M., Necoechea-Mondragón H., Hernández-Sánchez H., Gutiérrez-López G.F. (2021). Surface roughness and textural image analysis, particle size and stability of microparticles obtained by microfluidization of soy protein isolate aggregates suspensions. Rev. Mex. Ing. Quim..

[45] Ding X., Li A., Yang F., Sun K., Sun X. (2020). β-Tricalcium phosphate and octacalcium phosphate composite bioceramic material for bone tissue engineering. J. Biomater. Appl..

[46] Wei S., Ma J.X., Xu L., Gu X.S., Ma X.L. (2020). Biodegradable materials for bone defect repair. Mil. Med. Res..

[47] Hu X., Wang T., Li F., Mao X. (2023). Surface modifications of biomaterials in different applied fields. RSC Adv..

[48] Migita S., Sato M. (2025). Protein adsorption and cell adhesion on metallic biomaterial surfaces. Adhesives.

[49] Nitti P., Narayanan A., Pellegrino R., Villani S., Madaghiele M., Demitri C. (2023). Cell-tissue interaction: The biomi-metic approach to design tissue engineered biomaterials. Bioengineering.

[50] Hamraoui A. (2025). Cell adhesion and surface interactions: A comprehensive review of surface energy, wettability, and topography effects. AIP Adv..

[51] Liu Y., Zhang T., Li M., Ouyang Z., Gao F., Liu C., Li C., Liu D., Zhou Z. (2021). PLGA hybrid porous microspheres as human periodontal ligament stem cell delivery carriers for periodontal regeneration. Chem. Eng. J..

[52] Guo X., Wu X., Sun Z., Li D., Jia H., Zhang K., Zhao Y., Zheng H. (2025). Preparation, characterization, and binding mechanism of pH-driven gliadin/soy protein isolate nanoparticles. Food Res. Int..

[53] Jiang J., Shi L., Ren Z., Weng W. (2023). Preparation and characterization of soy protein isolate films by pretreatment with cysteine. Food Chem. X.

[54] Wang S., Lu Y., Ouyang X.K., Ling J. (2020). Fabrication of soy protein isolate/cellulose nanocrystal composite nanoparticles for curcumin delivery. Int. J. Biol. Macromol..

[55] Li C., He M., Tong Z., Li Y., Sheng W., Luo L., Tong Y., Yu H., Huselstein C., Chen Y. (2016). Construction of biocompatible regenerated cellulose/SPI composite beads using high-voltage electrostatic technique. RSC Adv..

[56] Zhang L., Xiao Q., Wang Y., Zhang C., He W., Yin L. (2017). Denatured protein-coated docetaxel nanoparticles: Alterable drug state and cytosolic delivery. Int. J. Pharm..

[57] Limsukon W., Rubino M., Rabnawaz M., Lim L.T., Auras R. (2023). Hydrolytic degradation of poly(lactic acid): Unraveling correlations between temperature and the three phase structures. Polym. Degrad. Stab..

[58] Moyo M.T.G., Adali T., Edebal O.H. (2024). ISO 10993-4 compliant hemocompatibility evaluation of gellan gum hybrid hydrogels for biomedical applications. Gels.

[59] Ji H., Zhang Z., Wang C., Li X., Zhang G., Liu D. (2024). In vitro cytocompatibility of triclosan coated Polyglactin910 sutures. J. Mater. Sci. Mater. Med..

[60] (2017). Biological Evaluation of Medical Devices—Part 4: Selection of Tests for Interactions with Blood.

[61] (2009). Biological Evaluation of Medical Devices—Part 5: Tests for In Vitro Cytotoxicity.

[62] Kikuchi T., Ohira S., Yamaguchi H. (2025). Viable cell density as an indicator for dynamic feeding strategy in fed-batch and perfusion CHO cell culture. Sci. Rep..

[63] Szwed A., Kim E., Jacinto E. (2021). Regulation and metabolic functions of mTORC1 and mTORC2. Physiol. Rev..

[64] Xie Y., Lei X., Zhao G., Guo R., Cui N. (2023). mTOR in programmed cell death and its therapeutic implications. Cytokine Growth Factor Rev..

[65] Ma M., He W., Liu X., Zheng Y., Peng J., Xie Y., Meng H., Wang Y. (2022). Soybean protein isolate/chitosan composite microcarriers for expansion and osteogenic differentiation of stem cells. Compos. Part B Eng..

[66] Songkoomkrong S., Nonkhwao S., Duangprom S., Saetan J., Manochantr S., Sobhon P., Kornthong N., Amonruttanapun P. (2024). Investigating the potential effect of *Holothuria scabra* extract on osteogenic differentiation in preosteoblast MC3T3-E1 cells. Sci. Rep..

[67] Zhang Z., Nam H.K., Crouch S., Hatch N.E. (2021). Tissue nonspecific alkaline phosphatase function in bone and muscle progenitor cells: Control of mitochondrial respiration and ATP production. Int. J. Mol. Sci..

[68] Sekaran S., Vimalraj S., Thangavelu L. (2021). The physiological and pathological role of tissue nonspecific alkaline phosphatase beyond mineralization. Biomolecules.

[69] Izumiya M., Haniu M., Ueda K., Ishida H., Ma C., Ideta H., Sobajima A., Ueshiba K., Uemura T., Saito N. (2021). Evaluation of MC3T3-E1 cell osteogenesis in different cell culture media. Int. J. Mol. Sci..

[70] Koblenzer M., Weiler M., Fragoulis A., Rütten S., Pufe T., Jahr H. (2022). Physiological mineralization during in vitro osteogenesis in a biomimetic spheroid culture model. Cells.

[71] Bernar A., Gebetsberger J.V., Bauer M., Streif W., Schirmer M. (2023). Optimization of the alizarin red S assay by enhancing mineralization of osteoblasts. Int. J. Mol. Sci..

[72] López-González I., Zamora-Ledezma C., Sanchez-Lorencio M.I., Tristante Barrenechea E., Gabaldón-Hernández J.A., Meseguer-Olmo L. (2021). Modifications in gene expression in the process of osteoblastic differentiation of multipotent bone marrow-derived human mesenchymal stem cells induced by a novel osteoinductive porous medical-grade 3D-printed poly(ε-caprolactone)/β-tricalcium phosphate composite. Int. J. Mol. Sci..

